# MptpB Inhibitor Improves the Action of Antibiotics against *Mycobacterium tuberculosis* and Nontuberculous *Mycobacterium avium* Infections

**DOI:** 10.1021/acsinfecdis.3c00446

**Published:** 2023-12-12

**Authors:** Pablo Rodríguez-Fernández, Laure Botella, Jennifer S. Cavet, Jose Domínguez, Maximiliano G. Gutierrez, Colin J. Suckling, Fraser J. Scott, Lydia Tabernero

**Affiliations:** † School of Biological Sciences, Faculty of Biology, Medicine and Health, University of Manchester, Manchester Academic Health Science Centre, M13 9PT Manchester, U.K.; ‡ Host Pathogen Interactions in Tuberculosis Laboratory, The Francis Crick Institute, NW1 1AT London, U.K.; § Lydia Becker Institute for Immunology and Inflammation, University of Manchester, M13 9PT Manchester, U.K.; ∥ Institut d’Investigació Germans Trias i Pujol, CIBER Enfermedades Respiratorias (CIBERES), Universitat Autònoma de Barcelona, 08916 Barcelona, Spain; ⊥ Department of Pure and Applied Chemistry, University of Strathclyde, 295 Cathedral Street, G1 1XL Glasgow, U.K.

**Keywords:** MptpB, Mycobacterium tuberculosis, Mycobacterium avium, combination of antibiotics, LAMP-1 lysosomal marker, Galleria mellonella

## Abstract

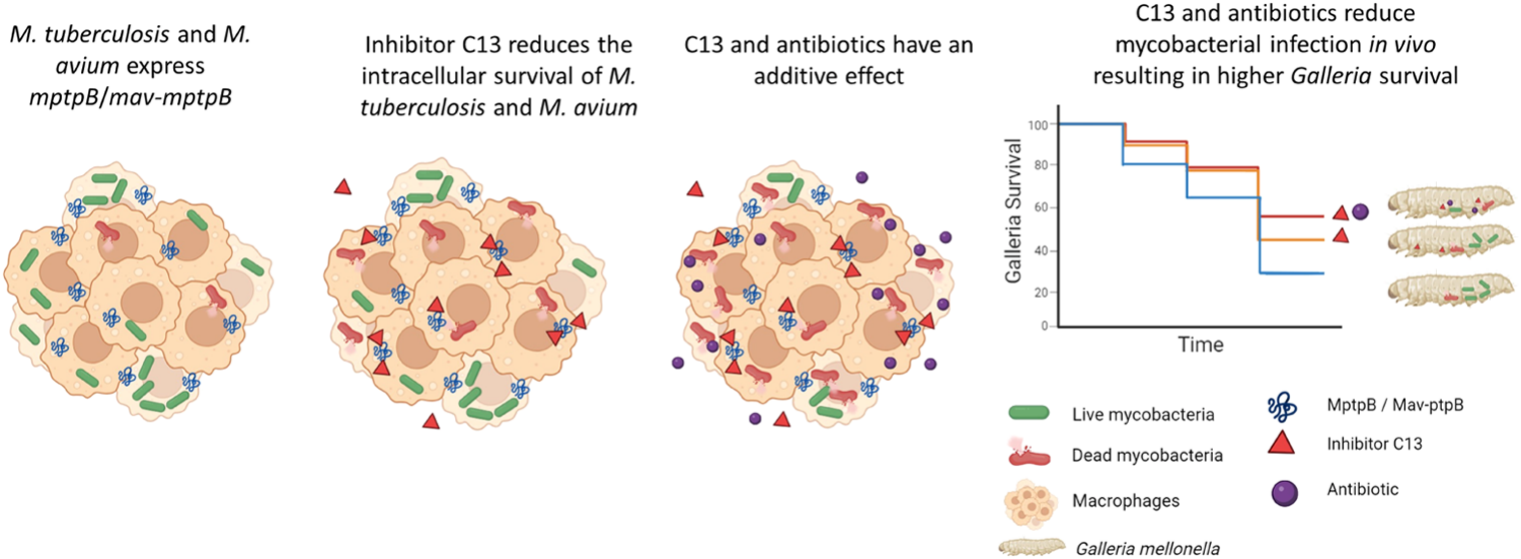

Treatment of *Mycobacterium tuberculosis* and *Mycobacterium avium* infections requires multiple drugs for long time periods. Mycobacterium protein-tyrosine-phosphatase B (MptpB) is a key *M. tuberculosis* virulence factor that subverts host antimicrobial activity to promote intracellular survival. Inhibition of MptpB reduces the infection burden *in vivo* and offers new opportunities to improve current treatments. Here, we demonstrate that *M. avium* produces an MptpB orthologue and that the MptpB inhibitor C13 reduces the *M. avium* infection burden in macrophages. Combining C13 with the antibiotics rifampicin or bedaquiline showed an additive effect, reducing intracellular infection of both *M. tuberculosis* and *M. avium* by 50%, compared to monotreatment with antibiotics alone. This additive effect was not observed with pretomanid. Combining C13 with the minor groove-binding compounds S-MGB-362 and S-MGB-363 also reduced the *M. tuberculosis* intracellular burden. Similar additive effects of C13 and antibiotics were confirmed *in vivo* using *Galleria mellonella* infections. We demonstrate that the reduced mycobacterial burden in macrophages observed with C13 treatments is due to the increased trafficking to lysosomes.

*Mycobacterium tuberculosis*, the causative agent of tuberculosis (TB), has been responsible for the death of over one billion people in just the last two centuries, more than any other infectious disease in history.^1^ It continues to be a leading cause of human mortality worldwide, being responsible for 1.5 million deaths every year.^2^ Treatment requires a minimum of 6 months with several antibiotics that can cause serious secondary effects like blindness or hepatotoxicity;^3^ but even more worrying is the rising number of untreatable, extensively drug-resistant TB cases.^2^ In addition, opportunistic infections with nontuberculous mycobacteria (NTM), such as *Mycobacterium avium*, are rising in developed countries due to comorbidity in patients with COPD, asthma, cystic fibrosis (CF), bronchiectasis, and HIV and particularly affecting people with immunodeficiencies and the elderly. As NTM are resistant to many antibiotics, these infections are extremely difficult and expensive to treat, requiring a minimum antibiotic treatment schedule of a year.^4^ Like TB, treatment of NTM infections requires multiple drugs, but success rates are lower and recurrence rates are higher than those for TB.

Over recent years, the use of antivirulence agents that target mycobacterial secreted virulence factors, as opposed to directly targeting bacterial growth, has gained interest as a new strategy to help clear infections.^5−7^ Antivirulence agents have been shown to successfully reduce the mycobacterial burden in both *in vitro* and *in vivo* infections.^8−14^ Antivirulence drugs could, in the future, be incorporated into current antimycobacterial treatment schedules as adjuvant therapies to increase efficacy and shorten treatment times. This would particularly benefit the treatment of drug-resistant mycobacterial infections that have a poor response to current antibiotics.

*M. tuberculosis* and *M. avium* are intracellular pathogens able to survive in macrophages by subverting phosphoinositide (PI) dynamics to prevent phagosome maturation, acidification, and fusion to lysosomes.^15,16^ PIs regulate many aspects of the endocytic pathway relevant to infection, including vesicle recycling, trafficking, autophagy, and lysosomal fusion.^17−19^ Specifically, PIs are involved in the recruitment of Early Endosomal Antigen 1 (EEA1) and GTPase proteins (Rab5 and Rab7), which are critical in controlling phagosome maturation and the clearance of the infection.^20,21^ One key lipid involved in phagolysosomal fusion is phosphoinositide-3-phosphate (PI3P), which is dephosphorylated by the secreted *Mycobacterium* protein tyrosine phosphatase B (MptpB), following *M. tuberculosis* phagocytosis.^11,22^ MptpB function not only inhibits the maturation of *M. tuberculosis*-containing phagosomes, preventing mycobacterial destruction in the lysosome, but also inhibits the innate immune response, decreasing IL-6, which impairs the activation of the systemic immune response.^23^ MptpB also decreases macrophage apoptotic activity, which plays a key role in activating the immune system.^23^ MptpB activity is, therefore, key for the viability of the bacteria inside host cells, and consistently, its inhibition or genetic disruption impairs the ability of *M. tuberculosis* to survive in macrophages or animal models of infection.^11,12,24^ Moreover, we have previously demonstrated that an MptpB inhibitor (C13) prevents PI3P dephosphorylation, significantly extending the presence of PI3P on *M. tuberculosis* phagosomes after infection.^11^

It is also reported that deletion or inhibition of one, two, or three mycobacterial phosphatases (MptpA, MptpB, or SapM) reduces the intracellular burden of *M. tuberculosis*(10,11,23−27) or *Mycobacterium marinum*,^28^ impairing their ability to replicate intracellularly. Consistently, overexpression of MptpB in macrophages enhances *M. tuberculosis* survival.^29^ Similarly to *M. tuberculosis*, levels of PI3P appear crucial for phagosome maturation during *M. avium* infections.^30^ Some studies have reported that *M. avium* is also able to produce secreted virulence factors like tyrosine phosphatase MptpA^31^ or protein kinase G.^32^

While the efficacy of antivirulence agents targeting MptpB in reducing the mycobacterial burden in macrophages and *in vivo* has been confirmed for both *M. tuberculosis* and the vaccine strain *Mycobacterium bovis* Bacille Calmette-Guérin (BCG),^10,11,22^ their ability to control infections by NTMs such as *M. avium* is not known.

Here, we confirm that a gene encoding an orthologue of MptpB (hereafter termed *mav-ptpB*) is present and expressed in an *M. avium* clinical isolate. We also demonstrate that the MptpB inhibitor C13 reduces the intracellular burden of *M. avium* in a way similar to that of *M. tuberculosis*. Furthermore, while previously we showed that MptpB inhibitors increase the killing efficacy of the first-line TB antibiotics rifampicin (RIF) and isoniazid (INH),^11^ here we show that treatment using C13 in combination with RIF or bedaquiline (BDQ) increased their efficacy by 25–50% compared to the antibiotic alone for both *M. avium* and *M. tuberculosis*. We also observed similar increased efficacy by combining C13 with two novel minor groove-binding (MGB) compounds from the Strathclyde MGB (S-MGB) family. S-MGBs are analogues of the natural product distamycin, which target multiple sites on bacterial DNA and are active against *M. tuberculosis*.^33,34^ We demonstrate that decrease in intracellular mycobacteria corresponds with a greater association of mycobacterial phagosomes with lysosomal markers. Finally, the efficacy of C13 alone and in combination with the antibiotics and compounds was also demonstrated in an *in vivo* model of infection using larvae of the waxworm *Galleria mellonella*.

## Results and Discussion

### *M. avium* Possesses and Expresses a *mptpB* Gene

Previously, we identified a large family of microbial atypical phosphatases related to MptpB, which, in addition to mycobacterial species, are present in many human pathogens, including fungi and bacteria.^22,35^ However, there is no reported characterization of these proteins in NTM species. *M. avium* is the most prevalent NTM in lung diseases, and its worldwide prevalence is increasing.^36^ We hypothesized that if an MptpB protein is expressed by *M. avium*, our MptpB inhibitor C13,^11^ which reduces *M. tuberculosis* infection *in vivo*, may also have efficacy against *M. avium*, *thus* offering another therapeutic application.

**Figure 1:**
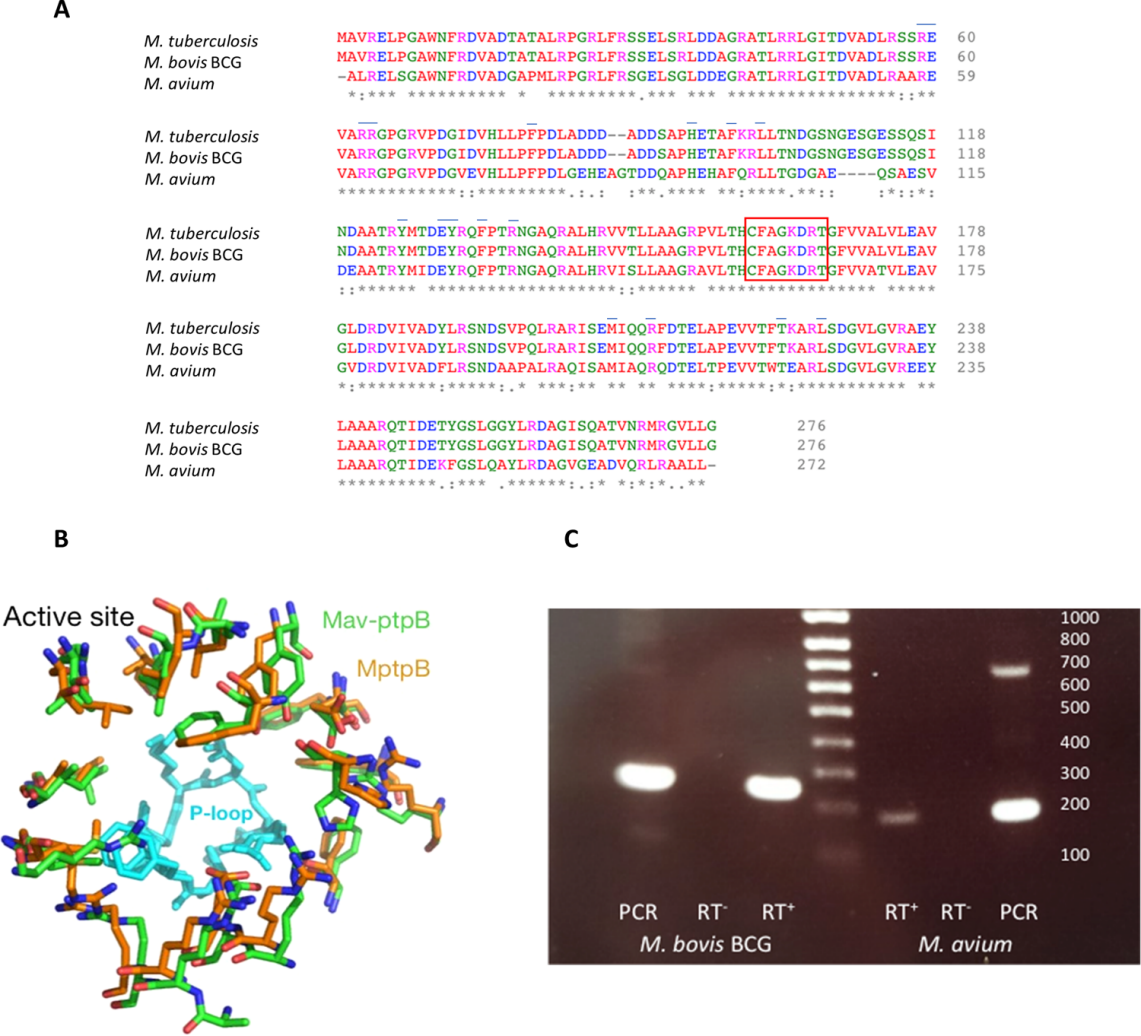
MptpB is conserved in *M. avium*. (A) Alignment of MptpB sequences of *M. tuberculosis* H_37_Rv (NP_214667.1), *M. tuberculosis* var. bovis BCG (WP_003401010.1), and *M. avium* (WP_009979776.1), shows 75% identity and 84% similarity for *M. tuberculosis* complex *vsM. avium* proteins, the boxed area indicates the active site signature. Under the blue line are shown conserved residues in the active site important for ligand binding.^11,37^ Alignments were prepared with the ClustalX format citation with Omega.^38^ In each column, “*” indicates identical residues, “:” conserved residues, and “.” semiconserved residues. (B) Superposition of the active site structure of *M. tuberculosis* MptpB, orange backbone (PDB ID: 2OZ5), and the Alphafold model^39^ of Mav-ptpB, green backbone. The signature P-loop at the bottom of the active site is shown in cyan. Residues shown have been reported to interact with inhibitors of MptpB, including C13.^11,37^ (C) Products generated by PCR using a DNA template (PCR) and RT-PCR using RNA template (RT+) and with inclusion of a negative control i*n* which the reverse transcriptase is substituted for water, (RT−) for *mptpB* from *M. bovis* BCG (left) and *M. avium* (right), corresponding to the anticipated sizes of 267 nts and 172 nts, respectively. Note that larger amplicons (of lesser intensity) were also detected by PCR amplification using a DNA template and presumed to be nonspecific products associated with the relatively low annealing temperature (57 °C).

A search of the NCBI gene database (WP_009979776) identified the *mptpB* DNA sequence in *M. avium* (*mav-ptpB*) containing 811 bp with 80% similarity to 1081 bp *M. tuberculosismptpB*. The deduced 272 amino acid protein sequence has 75% identity and 84% similarity to the 276 amino acid MptpB produced by *M. tuberculosis* or *M. bovis* BCG (Figure 1A). Furthermore, the active site signature, CFAGKDRT (P-loop motif), is strictly conserved in all three mycobacterial proteins, *M. tuberculosis*, *M. bovis* BCG, and *M. avium* (Figure 1A). The P-loop contains the catalytic Cys, Asp, and Arg residues previously identified in MptpB and orthologues.^22,35^ In addition, the residues lining the active site and reported to participate in ligand binding interactions are also conserved in Mav-ptpB (Figure 1A, B).^11,37^

Next, we investigated whether the *mav-ptpB* gene is present and expressed in an *M. avium* clinical isolate. Polymerase chain reaction (PCR) was used to amplify part of the gene from *M. avium* and *M. bovis* BCG DNA, with the generated products being of the anticipated sizes for *mav-ptpB* (172bp) and *mptpB* (267pb), thus confirming its presence (Figure 1B). Gene expression was subsequently confirmed by RT-PCR with RT-dependent products detected with both *M. avium* and *M. bovis* BCG RNA (Figure 1B). Figure S-1 shows the binding site of the primers used and the alignment of three Mav-ptpB sequences compared to MptpB.

### MptpB Inhibitor C13 Reduces the Intracellular Survival of *M. avium*

Having confirmed the presence and expression of *mav-ptpB*, next we tested if the MptpB inhibitor C13 would reduce the survival of *M. avium* in infected macrophages. Previously, we showed that C13 reduces the intracellular burden of *M. bovis* BCG and *M. tuberculosis* (drug-sensitive and multidrug-resistant strains) in macrophages,^11^ and hence, *M. tuberculosis* was included as a control for these experiments. Macrophages were infected with *M. tuberculosis* or *M. avium* (MOI 1:1) and treated with inhibitor C13 (29 μg/mL to maintain consistency with our previous publication^11^). RAW264.7 macrophages are a well-established *in vitro* model for *M. avium* infection studies, and since the survival of *M. avium* in THP-1 macrophages is substantially reduced compared to in RAW264.7 after 1 day,^40,41^ RAW264.7 macrophages were used for all infections with *M. avium*. In contrast, human-derived THP-1 macrophages are commonly used in models of *M. tuberculosis* infections and were consistently used for all *M. tuberculosis* assays.

**Figure 2:**
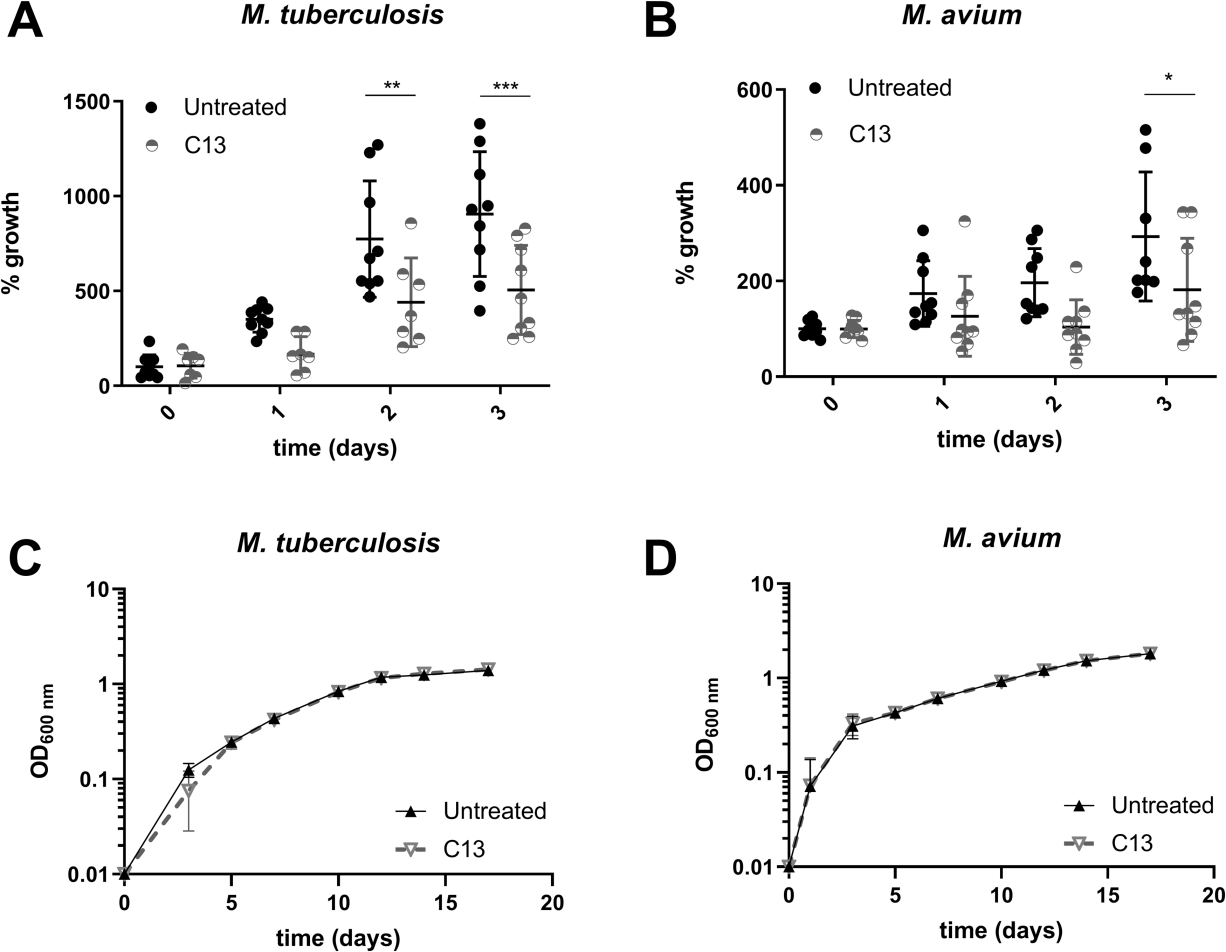
C13 reduces the intracellular *M. avium* burden without affecting the extracellular bacterial growth.

The bacterial burden was monitored by determining the mycobacterial colony forming units (CFU), following lysis of infected macrophages daily, up to 3 days post-infection. Treatment with C13 resulted in a tendency to a lower bacterial burden (% growth) detectable from 1 day post-infection, with a significant reduction of 44% (*p* = 0.0006) and 38% (*p* = 0.0186) in the intracellular burden of *M. tuberculosis* and *M. avium*, respectively, compared to untreated, at 3 days post-infection (Figure 2A,B). The reductions are similar to our previously reported findings for *M. bovis* BCG and *M. tuberculosis* with this inhibitor.^11^ The similar reduction in the *M. avium* infection burden suggests a role for Mav-ptpB similar to that of MptpB in promoting intracellular survival.

Effect of inhibitor C13 (29 μg/mL) on the intracellular growth of (A) *M. tuberculosis* in THP-1 macrophages or (B) *M. avium* in RAW264.7 macrophages up to 3 days post-infection. The data points show bacterial numbers recovered from macrophages at various time points as a percentage of those recovered at time (0) without C13 treatment. Data show technical replicates from three independent experiments with SD.

The effect of the inhibitor C13 (29 μg/mL) on the extracellular growth of (C) *M. tuberculosis* and (D) *M. avium* in Middlebrook 7H9 medium was monitored over 17 days by optical density at 600 nm (OD_600nm_). Data show the mean with SD of three technical replicates of at least two independent experiments. *p < 0.05, **p < 0.01, ***p < 0.001.

While C13 has been shown to have efficacy in reducing *M. tuberculosis* or *M. bovis* BCG survival in macrophages and animal infection models, it has no direct effect on the extracellular growth of these mycobacteria, consistent with the role of the secreted MptpB in intracellular survival, but not essential for growth.^10,11,42^ Similarly, we show here that C13 did not affect the extracellular growth of *M. avium* over the course of 17 days in comparison to untreated bacteria (Figure 2C,D), consistent with Mav-ptpB being required for the intracellular, but not extracellular, growth of *M. avium*. Overall, these results suggest that the mechanism of action of MptpB phosphatase as an intracellular survival factor is conserved in *M. avium*.

### Combination of C13 with Antibiotics Has Additive Effects in Reducing the Intracellular Mycobacterial Burden

Previously, we have seen enhanced efficacy when combining C13 and first-line antibiotics RIF (0.3 μg/mL) and INH (0.1 μg/mL) in reducing the intracellular burden of *M. bovis* BCG in mouse J774 macrophages.^11^ Next, we wanted to investigate if combinations of C13 with newly approved mycobactericidal antibiotics, BDQ and pretomanid (PRT), or example novel antimycobacterial compounds at the drug discovery phase, specifically Strathclyde Minor Groove Binders (S-MGBs, S-MGB-362, and S-MGB-363), would also show additional efficacy in reducing the intracellular burden, similarly to RIF.^34^Figure S-2 shows the structures of C13, S-MGBs, and antibiotics used in this study.

RIF is a very effective antibiotic against *M. tuberculosis*, with bactericidal activity. RIF is also a recommended antibiotic for the treatment of *M. avium*,^4^ although the inhibition of the β-RNA polymerase by RIF in slower-growing MAC species may result in bacteriostatic rather than bactericidal effects.^43^ BDQ and PRT have been recently introduced for the treatment of MDR-TB, and WHO guidelines recommend including BDQ in all regimens for the treatment of RIF-resistant strains.^44^ BDQ may also be recommended for the treatment of *M. avium* infections in patients who are intolerant to conventional antibiotics.^4^ PRT is included in all shortened 6–9 months treatments against MDR-TB.^44^

We first examined the cytotoxicity of a range of concentrations of RIF, BDQ, and PRT (0.625–80 μg/mL) in RAW264.7 macrophages (Figure S-3A). We selected 4 μg/mL as the highest antibiotic dose to be used in future experiments since BDQ 5 μg/mL reduced macrophage viability by 24.3% (Figure S-3A). We did not observe cytotoxicity associated with the S-MGBs in THP-1 at any of the concentrations tested (Figure S-3B) and therefore selected the concentration of 2.9 and 3.3 μg/mL (4 μM) for S-MGB-362 and S-MGB-363, respectively (Table S-1), as these concentrations have been reported to reduce the mycobacterial burden of infected cells by half.^33,34^

The intracellular burden on day 3 post-infection of (A) *M. tuberculosis* and (B) *M. avium* untreated or treated with the indicated antibiotics in the presence of inhibitor C13 (29 μg/mL) as a percentage of the bacterial burden with the same treatment but in the absence of C13. Concentrations of antibiotics are shown in μg/mL. Data show the mean with SD of three technical replicates of at least three independent experiments. (C) Heat map showing the percentage of additional reduction in the bacterial burden when adding C13 with the antibiotics compared to the same antibiotic without the inhibitor on day 3 post-infection, with the control showing the percentage reduction when adding C13 compared to no treatment. Green values indicate the greatest reductions. *p < 0.05, **p < 0.01, ***p < 0.001, ****p < 0.0001.

To compare with our previous results combining C13 with RIF,^11^ we also selected a dose of 0.3 μg/mL to be used for RIF, BDQ, and PRT. Although inhibitor C13 had previously shown no cytotoxic effects even at concentrations as high as 181.3 μg/mL,^11^ we assessed the potential cytotoxicity of the combinations of all compounds with C13 at 29 μg/mL in RAW264.7 macrophages and THP-1 macrophages. No cytotoxicity was observed for any of the combinations selected (Figure S-3B–D).

Having established the antibiotic and C13 levels for use, macrophages were infected with *M. avium* or *M. tuberculosis* and treated for 3 days with combinations of antibiotics (RIF, BDQ, or PRT) at concentrations of 0.3 or 4 μg/mL with or without C13 (29 μg/mL).

**Figure 3:**
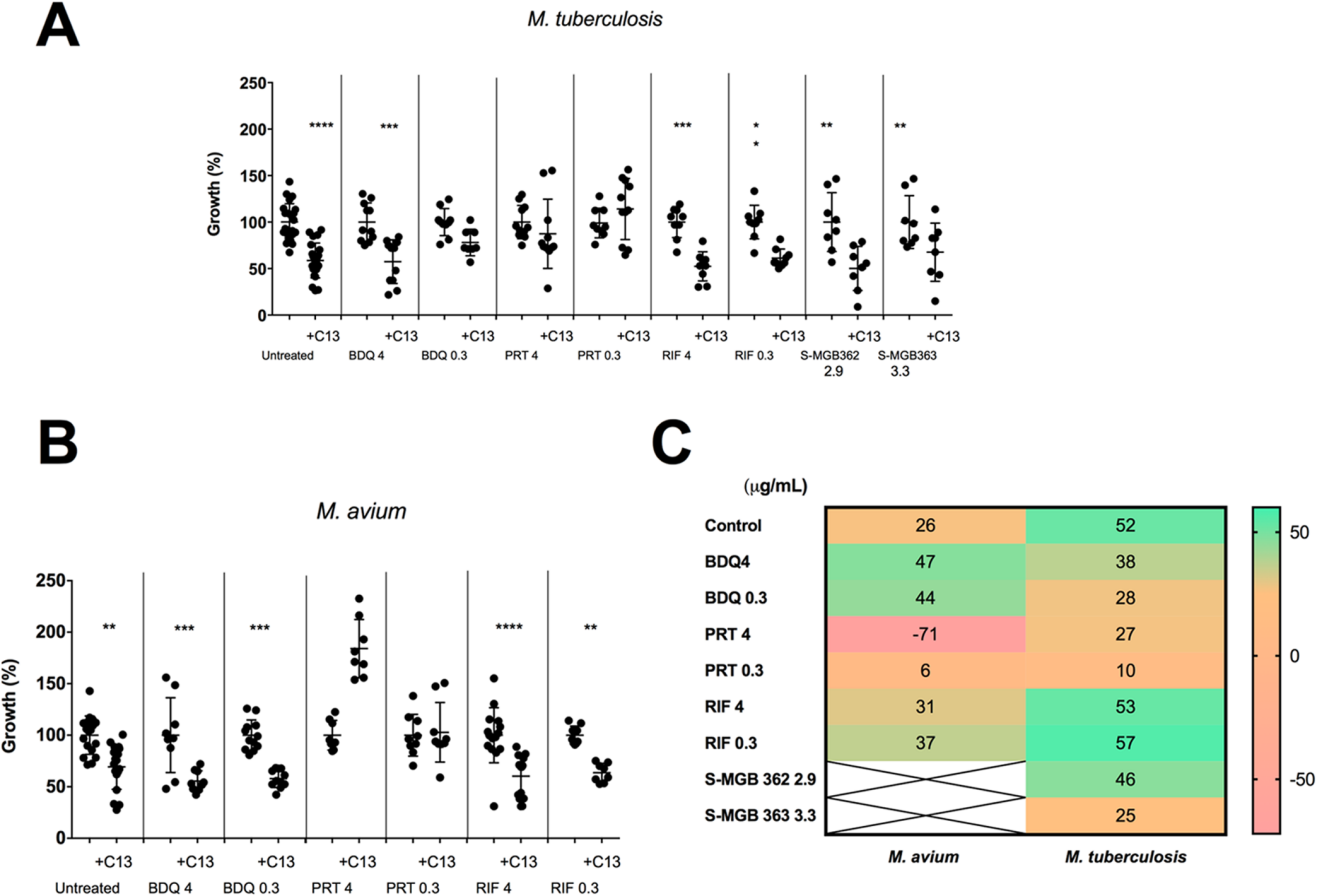
Combination of C13 with antibiotics reduces the mycobacterial intracellular burden compared to that with antibiotics alone.

Compared to the antibiotic treatment alone, we observed that the addition of the MptpB inhibitor caused an additional reduction of 25–50% in the bacterial burden for both *M. avium* and *M. tuberculosis* (Figure 3A,B), except for PRT, where we observed no reduction.

**Figure 4:**
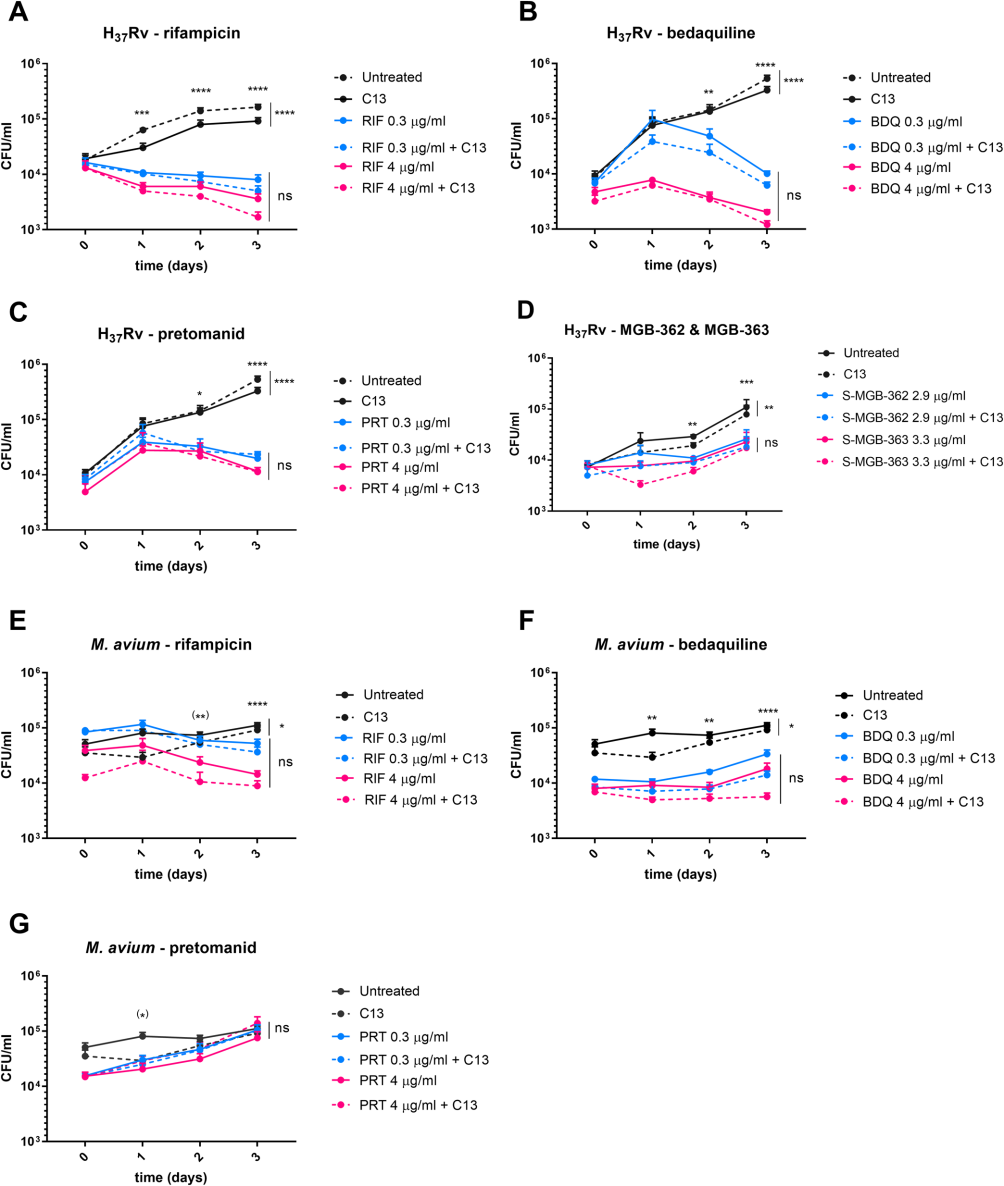
Effect of C13 and antibiotics in the intracellular burden of mycobacteria. Intracellular CFU is obtained from infections with *M. tuberculosis* (A–D) or *M. avium* (E–G). Data show the mean with SEM of three technical replicates of at least three independent experiments. Upper stars show statistical significance of antibiotic combinations compared to the control; if stars are in brackets, only the highest concentration is significantly different, where *p < 0.05, **p < 0.01, ***p < 0.001, ****p < 0.0001.

The best combination for reducing *M. tuberculosis* numbers was C13 + RIF (Figure 3C). Treatment with any of the antibiotics alone effectively reduced the *M. tuberculosis* intracellular burden from day 1, reducing by up to 2.4 logs on day 3 (Figure 4A–C). Treatment with C13 furthered the reduction by up to 0.2 logs on day 3 (*p* < 0.0001), equivalent to a 56.7% lower intracellular burden (Figure 3C).

For *M. avium* infections, the best combination was C13 + BDQ (Figure 3C). BDQ and RIF effectively reduced the intracellular burden by up to 0.9 logs on day 3 (Figure 4D,E). In contrast to *M. tuberculosis*, we observed that BDQ has bacteriostatic rather than bactericidal effects in *M. avium*, and PRT was not even bacteriostatic (Figure 4F,G), as previously described.^4,45^ Treatment with C13 in addition to BDQ resulted in a further reduction of up to 0.1 log on day 3 (*p* < 0.03), which is a 47.1% lower intracellular burden than with BDQ alone (Figure 3C).

Although the antibiotic that gave the best additive effect with C13 differed between *M. tuberculosis* and *M. avium*, 4 μg/mL BDQ with the C13 inhibitor was the combination that was most effective in reducing the intracellular burden of both species.

The S-MGBs, at 2.9–3.3 μg/mL, had a lesser effect in reducing the intracellular *M. tuberculosis* burden (Figure 4D). A potential explanation is that the MIC for S-MGBs is higher than for the other antibiotics (Table 1) and that for S-MGB-362; 2.9 μg/mL is reported to be the MIC_50_ for *M. tuberculosis* cell-culture infections,^34^ while RIF 0.3 is greater than MIC_90_ (Figure S-3A). Nevertheless, the addition of C13 to S-MGBs caused similar reductions in *M. tuberculosis* numbers as observed when adding C13 to antibiotics (Figure 4A,C).

**Table 1:** Minimal Inhibitory Concentrations (MICs) of the Antibiotics Used in This Study and Values in Combination with the C13 MptpB Inhibitor

MIC [REMA] [μg/mL]		+C13 (29 μg/mL)
*M. tuberculosis* H37Rv		
RIF	0.064	0.064
BDQ	0.064	0.064
PRT	0.064	0.064
S-MGB-362	0.29	0.29
S-MGB-363	0.33	0.33
*M. tuberculosis* Δ*mptpB*		
RIF	0.064	0.064
BDQ	0.064	0.064
PRT	0.064	0.064
*M. tuberculosis* Δ*mptpB:mptpB*		
RIF	0.064	0.064
BDQ	0.064	0.064
PRT	0.064	0.064
*M. avium*		
RIF	0.512	0.512
BDQ	0.016	0.016
PRT	32	32

### C13 Has No Effect on Antibiotic Efficacy against Extracellular Mycobacteria

Next, we tested if the reduction in the intracellular bacterial burden observed by treatment with C13 in combination with the antibiotics compared to antibiotic treatment alone was due to the inhibitor decreasing the MICs of the antibiotics or rather an additive effect. We first determined the MICs of antibiotics RIF, BDQ, and PRT for *M. tuberculosis* and *M. avium*. The PRT MIC for *M. avium* was much higher than for *M. tuberculosis* (32 *vs* 0.064 μg/mL) (Table 1), consistent with no significant reduction in the *M. avium* burden of macrophages by treatment with 0.3 or 4 μg/mL PRT (Figure 3B) and the high MIC values of PRT reported in the literature.^46^ The RIF MIC for *M. avium* was 0.512 μg/mL, eight times higher than for *M. tuberculosis*, whereas *M. avium* was more sensitive to BDQ (MIC of 0.016 *vs* 0.064 μg/mL) (Table 1).

To exclude the possibility that C13 may increase or decrease the effects of the antibiotics used in the combinations, we determined the MIC values for all antibiotics in the presence of C13 against extracellular bacteria. No differences in the MIC values were detected in the presence or absence of C13 (Table 1). Consistent with these results, there was no change in the MIC values associated with *mptpB* deletion in *M. tuberculosis* (strain Δ*mptpB*) or in the MptpB complemented strain Δ*mptpB:mptpB* (Table 1).

Taken together, our data show that when the C13 inhibitor is combined with current anti-TB antibiotics, there is an additive effect in reducing the intracellular infection burden. Although similar additive effects are seen with RIF and BDQ, there is no additive effect with PRT. These data suggest that MptpB inhibitors may serve as potential adjuvants to the antibiotic treatment of infections.

### C13 Decreases the Intracellular Mycobacterial Burden by Increasing Trafficking to Lysosomes

We have already demonstrated that inhibition of MptpB changes the association of PI3P with the mycobacterial phagosome,^11^ PI3P being a critical PI in the regulation of the endosomal pathway and fusion to lysosomes. Thus, we hypothesized that MptpB inhibition may promote phagolysomal fusion, leading to subsequent bacterial killing by exposure to the lysosomal antimicrobial activity.

To test this hypothesis, we infected macrophages with *M. tuberculosis* or *M. avium*, treated with antibiotics and/or C13, and evaluated the colocalization of the lysosomal-associated membrane protein-1 (LAMP-1) with the bacteria 1 day post-infection by fluorescence microscopy.

**Figure 5:**
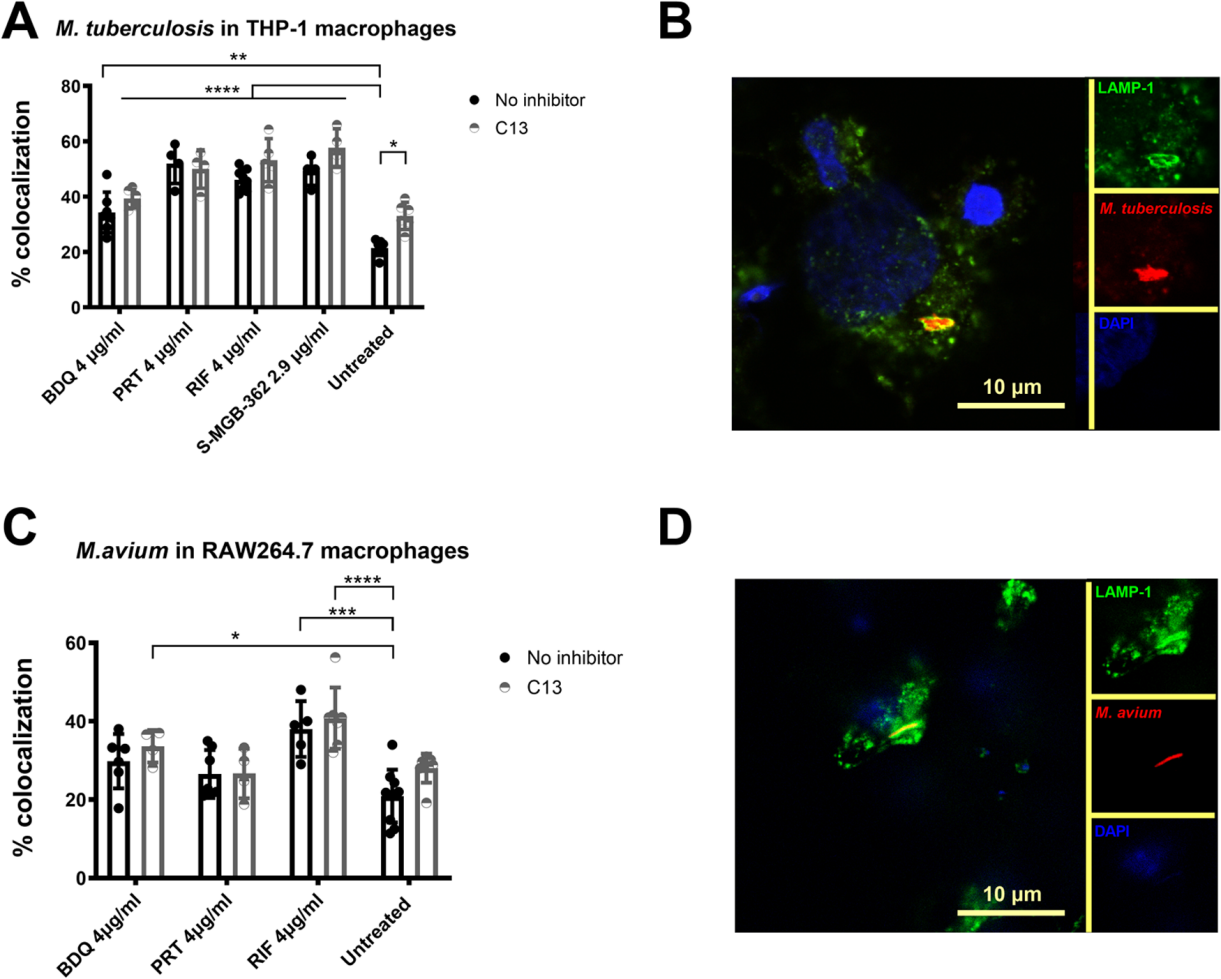
C13 increases mycobacterial trafficking to lysosomes. (A, C) Percentage of colocalization of LAMP-1 with *M. tuberculosis* (A, B) or *M. avium* (C, D) containing phagosomes in infected THP-1 or RAW264 macrophages, respectively, in the presence or absence of inhibitor C13 (29 μg/mL) with and without antibiotic treatment. Significant differences compared to untreated (without C13 inhibitor) are shown in the graph, where *p < 0.05, **p < 0.01, ***p < 0.001, ****p < 0.0001. (B, D) Representative images of positive colocalization (bacteria expressing mCherry are red, LAMP-1 labeled with Alexa488 is green, DAPI for nuclei). Data show the mean with SD of at least two technical replicates of three independent experiments.

Compound C13 alone significantly increased (by 12%) the association of LAMP-1 with *M. tuberculosis-*containing phagosomes (*p* = 0.0115) compared to untreated. A tendency to higher levels of colocalization (8%) of LAMP-1 with *M. avium*-containing phagosomes was also detected with C13 treatment, although this was not significant (Figure 5). These data are consistent with C13 treatment increasing mycobacterial trafficking to lysosomes.^27^

Treatment of infected macrophages with antibiotics and S-MGB-362 also increased the colocalization of *M. tuberculosis* with LAMP-1 by 13–31% (*p* < 0.0019) compared to untreated (Figure 5A), with BDQ showing the least increase. However, it is unlikely that the antibiotics directly enhance trafficking to lysosomes; rather, their bactericidal activity causes an increase in the proportion of dead bacteria, which are thus unable to deploy the weaponry to disrupt phagosome maturation. Indeed, Giraud-Gatineau et al. have previously reported that although the antibiotic BDQ is able to upregulate the lysosomal pathway triggering phagosome-lysosomal fusion, RIF is not.^47^

For *M. avium*, the increased colocalization of bacteria with lysosomes following antibiotic treatment was less substantial than with *M. tuberculosis*, especially in the case of PRT, and showed practically no increase in association (5%) compared to the controls (Figure 5C). These results are consistent with PRT being ineffective at the concentrations used in our infection experiments with *M. avium*.

A tendency for increased colocalization of both *M. avium* and *M. tuberculosis* with lysosomes was observed when C13 and antibiotic treatments were combined, except for PRT (Figure 5A,C), correlating with the lack of an additive effect for C13 with PRT in reducing the intracellular bacterial burden. We suggest that the RIF, BDQ and S-MGBs mechanisms of action in combination with MptpB result in different intracellular outcomes compared to PRT, an antibiotic that blocks a cell wall biosynthesis enzyme. Representative images of colocalization of mycobacteria with LAMP-1 are shown in Figure 4B,D.

The higher LAMP-1 colocalization with *M. tuberculosis* compared to *M. avium* following antibiotic treatment is also consistent with the antibiotics having a greater bactericidal effect against *M. tuberculosis* than against *M. avium* in our infection experiments.

Additionally, S-MGB-363 showed a very bright autofluorescence, which enabled us to confirm that S-MGB colocalizes with the DNA of *M. tuberculosis*, as expected by its suggested mechanism of action (Figure S-4). While, fluorescent probe S-MGBs have been used to confirm DNA association in parasites and bacteria,^48,49^ this is the first example for mycobacteria. However, it was excluded from our LAMP-1 analyses, as the fluorescence interfered with the detection of the marker fluorescence.

### Combination of C13 with Antibiotics Reduces Bacterial Survival *In Vivo*

We next checked the additive antimycobacterial effect of the combination of C13 with antibiotics BDQ, RIF, and S-MGB-362 in *in vivo* experiments. *G. mellonella* (waxworm) larvae represent a novel infection model for *M. tuberculosis*, providing a rapid and affordable evaluation of drug efficacy.^50,51^ We used *G. mellonella* infected with *M. bovis* BCG (for biosafety reasons) or *M. avium* to evaluate the efficacy of treatment with BDQ, RIF, and S-MGB-362 alone, MptpB inhibitor C13, or a combination of both. Since the combination of PRT and C13 showed no additive effect in reducing the bacterial burden in macrophage infections and no differences in LAMP-1 colocalization, we excluded this antibiotic from the *in vivo* experiments.

First, we tested the effect of a range of concentrations of RIF and BDQ between 0.3 and 20 mg/kg on the survival of *G. melonella* infected with *M. bovis* BCG or *M. avium* (Figure S-5). All concentrations of antibiotics used caused an increase in the survival of the worms infected with *M. avium* (*p* < 0.0416), but a concentration of at least 4 mg/kg of any of them was required to significantly increase survival with *M. bovis* BCG infection. For comparison, we selected lower and higher doses of antibiotics to test in combination with C13 in the infections of *G. melonella* with both bacteria. Doses of RIF and BDQ of 0.3 and 4 mg/kg were selected because, at this concentration, the effect of the antibiotics is moderate; thus, any additive effects of C13 may be detected. Having established the concentrations of antibiotics to use, we adjusted the concentrations of C13 and S-MGB-362 by a similar magnitude as BDQ or RIF to make them comparable to the concentrations used in macrophage infections. All concentrations of antibiotics and combinations tested were well tolerated by the worms (Figure S-6).

**Figure 6:**
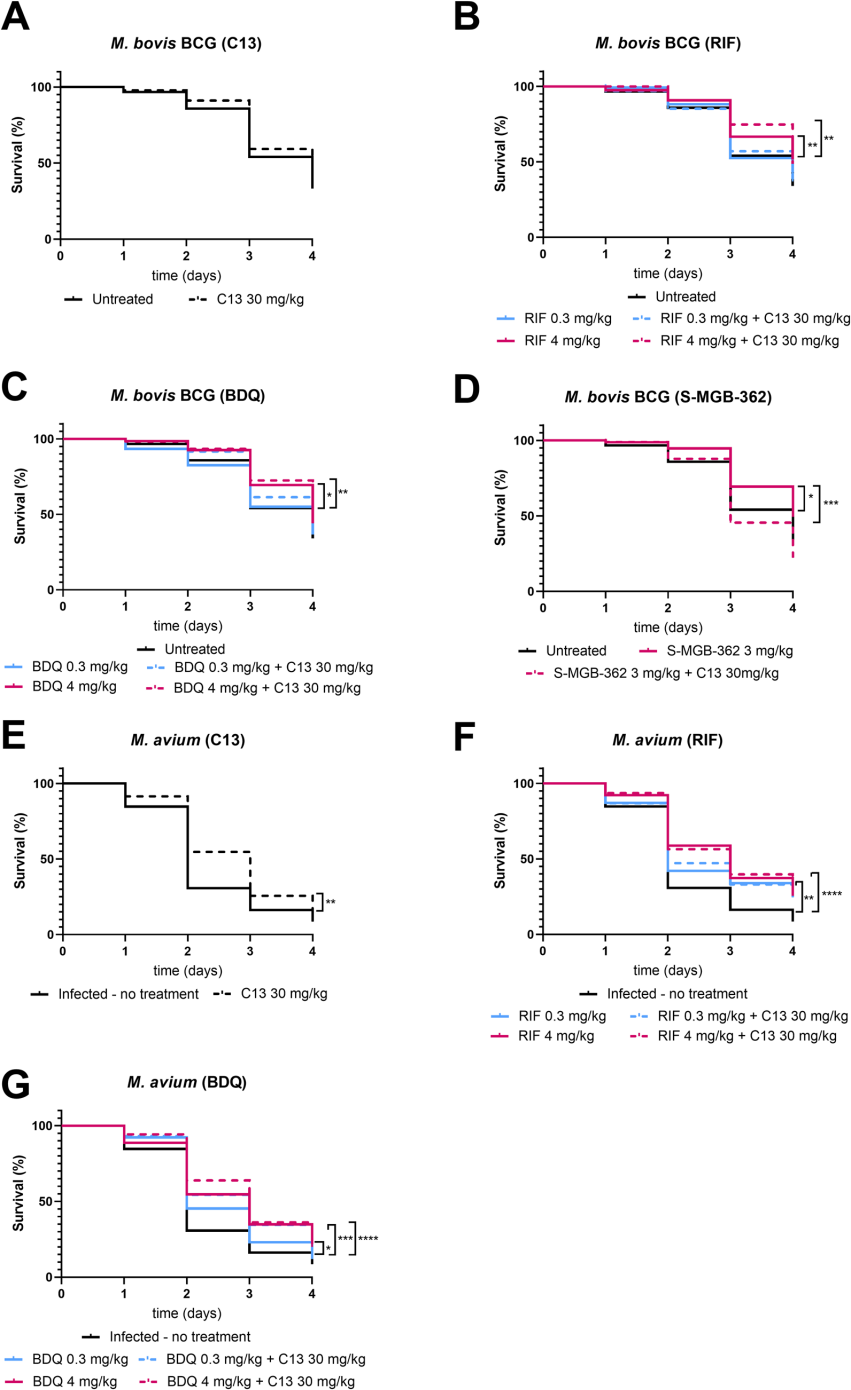
Kaplan–Meier survival curves of *G. mellonella* larvae infected with *M. bovis* BCG or *M. avium* and treated with C13 and/or antimycobacterial drugs. Larvae (*n* = 15 per group) were infected and treated in a 10 μL single injection. Survival was monitored daily over a period of 4 days. Data are from a minimum of five independent experiments. Data show the survival of *Galleria* infected with *M. bovis* BCG (A–D) or *M. avium* (E–G) when treated with C13 or the combinations of C13 with RIF, BDQ, or MGB, where *p < 0.05, **p < 0.01, ***p < 0.001, ****p < 0.0001.

Treatment of *M. bovis* BCG infections with C13 had a non-significant increase (5%) in the survival of *G. melonella* compared to no treatment (Figure 6A). Treatment with the higher dose of antibiotics BDQ and RIF significantly increased *G. melonella* survival as expected. Treatment with C13 in combination with the antibiotics increased survival between 5–10% of the infected *G. melonella* compared to treatment with antibiotics alone (Figure 6B–D), and combination with RIF resulted in the highest survival at 75%. While treatment with the compound S-MGB-362 (3 mg/kg) alone increased *G. melonella* survival similarly to the higher dose of antibiotics, interestingly, S-MGB-362 in combination with C13 appears to decrease *G. melonella* survival by 27% compared with the drug alone (*p* = 0.0039) (Figure 6B), suggesting antagonism between these compounds in this system.

For infections with *M. avium*, notably, the treatment with C13 alone significantly increased the survival (13.2%) of the wax worms compared to the untreated group (*p* < 0.0041) on day 4 and 25% on day 3 (Figure 6E). Combinations of C13 with BDQ and RIF on day 4 increased survival by 5–10%, as for BCG, with the most effective combination being C13 with BDQ at 0.3 or 4 mg/kg (Figure 6F,G).

**Figure 7:**
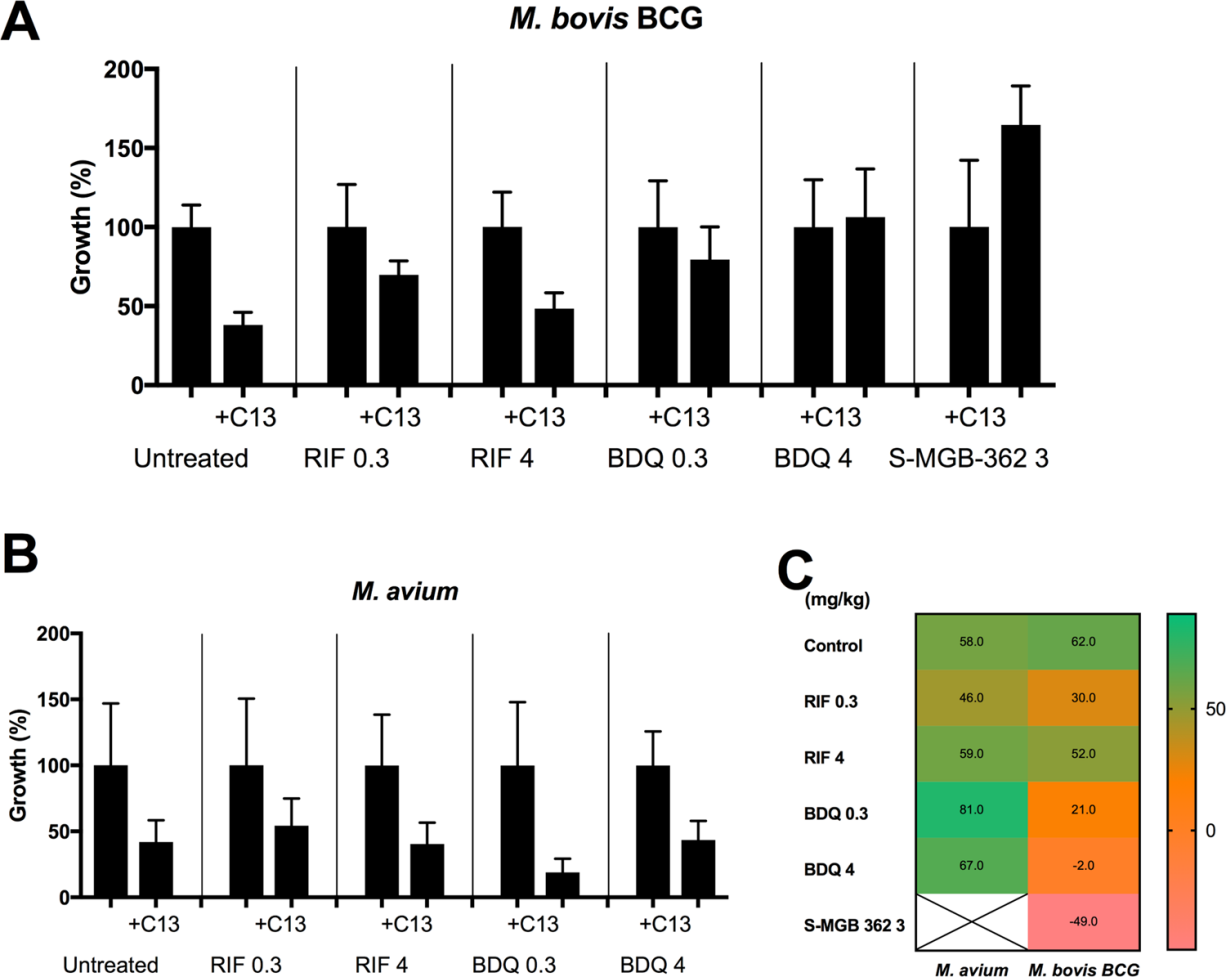
*M. bovis* BCG or *M. avium* burden in Galleria 4 days postinjection. The burden of *Galleria* on day 4 post-infection of (A) *M. bovis* BCG and (B) *M. avium* untreated or treated with the indicated antibiotics in the presence of inhibitor C13 (29 μg/mL) as a percentage of survival with the same treatment but in the absence of C13. Data show the mean with SD of at least three independent experiments. (C) Heat map showing the percentage of additional reduction in the bacterial burden (CFUs) when adding C13 with the antibiotics compared to the same antibiotic without the inhibitor on day 4 post-infection. Green values indicate the greatest reductions. Due to the variation of the data, no significant differences were observed.

To gain further insight into the effect of C13 and the antibiotics on *G. melonella* infected with *M. bovis* BCG or *M. avium*, we also analyzed the bacterial burden in the infected larvae. For this, live worms from the same experiments as above were homogenized on day 4; the homogenate was decontaminated with sodium hydroxide, and mycobacterial CFU was determined following plating onto 7H11 medium. Although differences are not statistically significant, we observe a trend in the reduction of the number of bacteria recovered from infected *G. melonella* when comparing treatments with C13 alone or combinations with antibiotics. The exceptions are C13 in combination with BDQ 4 mg/kg and S-MGB-362 3 mg/kg with *M. bovis* BCG. (Figure 7). Overall, the MptpB inhibitor C13 had the greatest additive effect in reducing the mycobacterial burden in *G. mellonella* when in combination with RIF (51.5% reduction) for *M. bovis* BCG infections, and in combination with BDQ for *M. avium* infections (81.2% reduction) (Figure 7C), confirming the observations from infected macrophages (Figure 3).

The mycobacterial burden from *G. mellonella* results show a similar overall trend to that observed from the survival curves (Figure 6), although a direct correlation is not observed, likely due to the irreversible impact of infection on the health of the worms (they all die beyond day 4). It is important to note that *Galleria*, although it is a more complex model than macrophages, is a very simplistic model that requires a high inoculum of *M. bovis* BCG,^50^ and it is not a natural host for mycobacteria.

However, we have shown that the reduction of the bacterial burden by inhibiting MptpB correlates well between infected worms and infected macrophages, showing C13 to be most effective in reducing the bacterial burden in combination with RIF for *M. bovis* BCG and BDQ for *M. avium* in both systems.

To the best of our knowledge, there is only one other reported *in vivo* study combining MptpB inhibitors with antibiotics in guinea pigs. In that study, Dutta et al.^12^ showed that inhibition of MptpA and MptpB together with isoniazid-rifampicin-pyrazinamide causes a reduction in bacterial numbers and improves lung histopathology.

## Conclusions

Overall, our data support that MptpB inhibitors may have good activity beyond tuberculous species, including *M. avium* and other related NTM pathogens. These species are difficult to treat because they have a plethora of escape mechanisms, such as inhibiting the maturation and acidification of phagosomes, inhibiting oxidative stress and the function of reactive oxygen and nitrogen intermediates, inhibiting apoptosis and autophagy, or even limiting lysosome formation.^15,52^ Despite lacking bactericidal activity, the MptpB inhibitor C13 is effective in reducing the bacterial burden in both macrophages and *G. melonella* infected with *M. bovis* BCG and *M. avium* and shows additive effect with antibiotics in the clinic RIF and BDQ, suggesting new effective combinations for treatment. Further studies in animal models of MptpB inhibitors with current antibiotics will help to refine the best combinations explored in this study and establish new regimens to improve current treatments, particularly for *M. avium* infections that have poor outcomes and high relapse.

## Materials and Methods

### Bacterial Strains and Cell Culture

THP-1 monocytes (ATCC, TIB-202) were cultured in RPMI-1640 medium (R8758-Sigma-Aldrich) and RAW264.7 macrophages (ATCC, TIB-71) were cultured in high-glucose DMEM (D-6429-Sigma-Aldrich), both containing l-glutamine supplemented with 10% heat-inactivated fetal bovine serum (FBS, Invitrogen) at 37 °C in 5% CO_2_. THP-1 was stimulated with 200 nM of phorbol 12-myristate-13-acetate (PMA, Sigma-Aldrich) O/N previous to any experiment.

*M. tuberculosis* strain H37Rv (ATCC) was used in all experiments. For the microscopy experiments, we used *M. tuberculosis* expressing wasabi or E2Crimson. The gene *mptpB (rv0153c)* was deleted from wild-type *M. tuberculosis* H37Rv using the ORBIT method.^53^ The transformants were verified by PCR, and the final clone was confirmed by WGS. Complementation of the deletion was achieved by expressing *mptpB* from the MS6 site in the mycobacterial chromosome under the control of the strong Phsp60 promoter.

*M. bovis* BCG Pasteur 1173p2 was used for DNA/RNA extraction and *Galleria* infections. For S-MGB-363 microscopy visualization, *M. bovis* BCG expressing GFP was used.

For *M. avium* infections, we used a clinical isolate from Germans i Pujol Hospital (Barcelona, Spain) and used this in all experiments. For microscopy sample preparation, we used *M. avium* (ATCC 700898) electroporated with the mCherry plasmid. All cultures were grown in Middlebrook 7H9 broth or on Middlebrook 7H11 agar (BD Diagnostics), both supplemented with 0.05% Tween 80, 0.2% glycerol, and 10% OADC (Oleic Albumin Dextrose Catalase) at 37 °C. Fluorescent strains were grown with kanamycin 50 μg/mL (GFP plasmid) or hygromycin 50 μg/mL (mCherry, wasabi, or e2crimson). All experiments with *M. tuberculosis* were carried out in a biosafety level 3 containment facility.

### Preparation of Drugs

BDQ (HY-14881/CS-2921) (CAS 843663–66–1, MedChemical Express) and PRT (PA-824) (CAS 187235–37–6, Adooq Bioscience) were prepared in dimethyl sulfoxide (DMSO) at 2 mg/mL. RIF (R-3501) (CAS13292–46–1, Sigma-Aldrich) was dissolved in methanol at 8 mg/mL. Zeocin (CL-990-cin) (CAS 11006–33–0, BioBasic) was prepared in Dulbecco’s phosphate-buffered saline (DPBS) at 12.5 mg/mL. Hygromycin (10687010) (CASInvitrogen) was used as directed (50 mg/mL in PBS). C13 (4-(3′,5′-dichloro-4′-hydroxy-3-biphenyl)-5-methylisoxazole-3-carboxylic acid) was dissolved in DMSO at 29 mg/mL and used at 29 μg/mL in experiments, to maintain consistency with our previous publication.^11^ S-MGB-362 and S-MGB-363 were kindly provided by Fraser J. Scott (University of Strathclyde, Glasgow) and were dissolved in DMSO at 4 mM.

### Genomic DNA Extraction, RNA Isolation, and DNA Amplification

Genomic DNA was extracted as previously reported, with some modifications.^54^ Briefly, a pellet from 50 mL of culture was incubated at 37 °C (shaking) in the presence of 0.5 mg of lysozyme (21560016–1, Bioworld). Then, sodium dodecyl sulfate (SDS) and proteinase K were added to final concentrations of 2% and 33 μg/mL, respectively, and volume was adjusted to 300 μL in 20 mM Tris/HCl pH 9. After 3h of incubation at 37 °C (shaking), 60 μL of 5 M NaCl was added, and 60 μL of sodium-chloride-Tris-EDTA (STE) buffer (100 mM NaCl, 1 mM EDTA, 10 mM Tris pH 8), and was incubated 15 min 65 °C. Then, the sample was mixed gently with 400 μL of phenol/chloroform/isoamyl 25:24:1 (P3803, Sigma-Aldrich) and centrifuged for 15 min at 11000*g*. The supernatant was transferred to a new tube, and the step of phenol/chloroform/isoamyl was performed. Then, 0.6 volumes of isopropanol were added to the aqueous supernatant and incubated for at least 30 min at −20 °C. After that, the sample was centrifuged for 10 min 11,000*g* and 0.5 mL of cold 75% ethanol was added to the pellet. The sample was again centrifuged for 10 min at 11,000*g* and the pellet was left dried. Finally, the pellet was resuspended in ultrapure water, and DNA concentration was measured on a Nanodrop 2000 (Thermo Scientific).

For RNA extraction, the same steps were followed as for DNA extraction but using acid phenol (9720, Ambion) instead of the phenol/chloroform/isoamyl mix. For a higher RNA purification, RNeasy columns were used (Quiagen); the sample was mixed with 350 μL of RLT (with 10 μL/ml β-mercaptoethanol), and then 295 μL of 95% ethanol was added. Mixed samples were then transferred to an RNeasy spin column and centrifuged for 15 s, and 350 μL of RW1 buffer was added and centrifuged for 15 s. Flow through was discarded, and 70 μL of RDD buffer with 4 IU of DNase I (M0303S, BioLabs) was added directly into the membrane. After 30 min of RT incubation, 350 μL of RW1 buffer was added and centrifuged for 15 s. After two washings of the membrane with 500 μL of RPE, RNA was eluted, and concentration was measured.

For cDNA synthesis, 1 μg of total RNA was added to 1.25 μL of 10 μM primer (*M. avium*: Forward: GGATTGGTGGTGGCGACGGTGCTC, Reverse: CTCGGTCCAGGTCACCAC; BCG: Forward: TAACCAATGGCGGGTCCAA, Reverse: GCAGGTAGTCGGCGACG) and incubated 5 min at 70 °C. Then, 1.25 μL of 10 mM DNTPs, 1 μL of M-MLV-RT (M1701, Promega), and 5× M-MLV-RT buffer and water were added according to a 25 μL reaction. The mix was incubated at 42 °C for 1 h.

For PCR amplification, 5× GC buffer, 0.75 μL of DMSO, 0.5 μL of 10 mM dNTPs, 10 μM of each primer, 0.3 IU of Phusion pol HF (K1031, APExBIO), 100 ng of DNA, and water up to 25 μL. Initial denaturation 98 °C 3 min, 4 cycles 98 °C 20 s, 60 °C 20 s, 72 °C 1 min, 20 cycles 98 °C 20s, 57 °C 20s, 72 °C 1 min, final extension 72 °C 6 min. Expected amplicons of 308nts for *M. avium* and 220 nts for BCG.

### Cytotoxicity Assays

A colorimetric assay using the tetrazolium dye 3-(4,5-dimethylthiazol-2-yl)-2,5-diphenyltetrazolium bromide (MTT) was performed as described previously.^27^ Briefly, 1.2 × 10^4^ THP-1 monocytes or 6 × 10^3^ RAW264.7 macrophages were seeded in flat-bottomed 96-well cell-culture plates (Corning). Compounds were added to the cells at 24 and 48h. At 72h, cell viability was assessed by adding 50 μL of MTT (M2128, Sigma-Aldrich) and incubating for 2 h at 37 °C in 5% CO_2_. Media was removed, followed by the addition of 200 μL of dimethyl sulfoxide (DMSO) and 25 μL of Sorensen’s glycine buffer (0.1 M glycine, 0.1 M NaCl, pH 10), and absorbance was measured at 570 nm. Each assay was performed in triplicates. A compound was considered toxic when macrophage viability was <70%.

### MIC Determination

Minimal inhibitory concentrations (MIC) of RIF (0.008–4.096 mg/mL), BDQ (0.008–0.256 mg/mL), PRT (0.008–32 mg/mL), S-MGB-362 (0.008–8.192 μM), and S-MGB-363 (0.008–8.192 μM) were tested as previously described.^55^

### Acellular Growth Curves

*M. tuberculosis* or *M. avium* was inoculated in flasks containing 25 mL of Middlebrook 7H9 with C13 (29 μg/mL) at a final OD_600 nm_ of 0.01. Controls were DMSO 0.1% only. Cultures were grown static over 17 days at 37 °C, and bacterial growth was monitored by OD at 600 nm. Experiments were performed in triplicate on at least two separate studies.

### Cell-Culture Infection Assays

Infection assays were done as previously reported.^27,55^ Briefly, 3 × 10^5^ THP-1 monocytes with 200 nM PMA or 6 × 10^4^ RAW264.7 macrophages were seeded in 24-well cell-culture plates in 500 μL of media and left resting with O/N. Then, the media was replaced with 300 μL of fresh media containing antibiotics, C13, and the bacteria for a final multiplicity of infection (MOI) of 1:1. After 4 h of infection, cells were washed 3 times with DPBS, and 500 μL of fresh cell media was added containing the antibiotics and/or C13 at 4 h, and again at 24h. At 1, 2, or 3 days post-infection, cells were lysed with 400 μL of ice-cold distilled water and, together with 100 μL of cell-pelleted supernatants, were plated onto 7H11 agar. All experimental points were plated as 10-fold dilutions in triplicate in at least three independent experiments. Colonies were counted after 15–25 days. A negative control of 0.1% DMSO was included.

### Indirect Immunofluorescence and Image Analysis

One × 10^5^ RAW264.7 macrophage or 2 × 10^5^ THP-1 macrophages were seeded on coverslips and rested overnight. Cells were then infected, as indicated. At 24h post-infection, cells were fixed with 4% methanol-free paraformaldehyde (PFA) (P6148, Sigma-Aldrich) in DPBS for 30 min. Coverslips were then quenched with 50 mM NH_4_Cl (044722, fluorochem) in PBS for 10 min at room temperature and permeabilized with DPBS containing 1% BSA, 0.05% saponine for 15 min. The primary antibody (24170, Abcam; 104B, Hybridoma Bank) was diluted in DPBS containing 1% BSA and incubated at 4C. The coverslips were washed 3 times in DPBS, and the secondary antibody was added in the same way as the primary antibody (anti-rat or -rabbit Alexa Fluor 488, Invitrogen) for 60 min at 4C. After three more washes with DPBS, nuclear staining was performed using 300 nM DAPI (Life Technologies, D3571) in DPBS for 10 min. One final wash with DPBS was performed before mounting the coverslips on glass slides using a Prolong Gold Antifade reagent mounting medium (P36934, Invitrogen).

For S-MGB-363 studies, *M. tuberculosis* expressing wasabi or uninfected THP-1 macrophages were added to a solution of 4 μM of S-MGB-363 for 2 h before intensive washing and fixing of the O/N in 4% PFA, and cells were stained with DAPI, as previously described, before mounting. Images were acquired on a Leica SP8 inverted microscope or a BX51 Olympus fluorescent microscope. Images were analyzed using the image analysis software ImageJ.

Images were analyzed using the image analysis software FIJI (US National Institutes of Health). Marker association with Mtb was analyzed as previously described.^56^ At least 100 bacteria per biological replicate of at least 3 independent experiments were analyzed during the analysis.

### *G. Mellonella* Survival Assay

*G. mellonella* larvae were purchased from Livefoods Direct Ltd. (Sheffield, U.K.). Larvae of 2–3 cm in length were infected with a final volume of 10 μL, containing the antibiotic (RIF 0.3 or 4 mg/kg, BDQ 0.3 or 4 mg/kg, S-MGB-362 3 mg/kg), inhibitor C13 (30 mg/kg), and bacteria (1.2 × 10^8^ CFU M. avium or 2.1 × 10^7^ CFU *M. bovis* BCG), into the hemocoel *via* the last proleg with a 30G needle. Infected larvae were incubated in the dark at 37 °C. Survival of infected larvae (*n* = 15 per group) following treatment was recorded every 24h for 96h. Larvae were considered dead when they failed to respond to touch. Control groups were infected with 10 μL of PBS-0.05% tween. Kaplan–Meier survival curves were plotted using data pooled from a minimum of three independent experiments.

To calculate the internal burden of bacteria, live worms at 96h post-infection were homogenized in a FastPrep-24 machine for 1 min to maximum potency in 2 mL tubes containing 800 μL of PBS-0.05% tween and 0.05 mL of 1 mm glass beads. Then, 300 μL of the sample was decontaminated with 150 μL of 1 M NaOH and amphotericin β to a final concentration of 50 μg/mL for 15 min. Samples were then centrifuged at maximum speed for 3 min, and the pellet was resuspended in 90 μL PBS-0.05% tween. All experimental points were plated onto 7H11 agar plates as 10-fold dilutions in triplicate with worms pooled from at least three independent experiments.

### Statistical Analysis

Statistical analysis was performed by using GraphPad Prism software. The definition of statistical analysis and *post hoc* tests used can be found in figure legends. The statistical significance of data is denoted on graphs by asterisks (*) where *p < 0.05, **p < 0.01, ***p < 0.001, ****p < 0.0001, or ns = not significant. ANOVA two-way analysis was performed for the intracellular assay analysis and long-rank (Mantel-Cox) test for survival curves of *Galleria*.

## Supporting Information Available

Equivalence of μg/mL and μM of non-commercial compounds used in this study (C13 and S-MGBs) (Table S-1); Mav-ptpB is conserved (Figure S-1); structure of the inhibitor C13, antibiotics RIF, BDQ and PRT, and S-MGBs used in this study (Figure S-2); viability of THP-1 or RAW264.7 macrophages three days after exposing to antibiotics alone or in combination with C13 measured by MTT assay (Figure S-3); image showing that S-MGB-363 colocalizes to the bacterial DNA (Figure S-4); Kaplan–Meier survival curves of *G. mellonella* larvae infected with BCG or *M. avium* and treated with C13 and or antimycobacterial drugs (Figure S-5); tolerability of compounds in *G. mellonella* uninfected larvae (Figure S-6) (PDF)
