# Supplementary material for: MptpB Inhibitor Improves the Action of Antibiotics against Mycobacterium tuberculosis and Nontuberculous Mycobacterium avium Infections
Source: ACS Infect Dis. 2023 Dec 12;10(1):170–83. doi: 10.1021/acsinfecdis.3c00446 (PMC10788870; doi:10.1021/acsinfecdis.3c00446)
Supplement: Supplementary file 1 — id3c00446_si_001.pdf [file id3c00446_si_001.pdf]

Supporting Information

An MptpB inhibitor improves the action of antibiotics against *Mycobacterium tuberculosis* and non-tuberculous *M. avium* infections

Pablo Rodríguez-Fernández[1], Laure Botella[2], Jennifer S. Cavet[1,3], Jose Domínguez[4] Maximiliano G. Gutierrez[2], Colin J. Suckling[5], Fraser J. Scott[5] and Lydia Tabernero[1,3*]

[1] School of Biological Sciences, Faculty of Biology, Medicine and Health, University of Manchester, Manchester Academic Health Science Centre, M13 9PT, Manchester, UK.

[2] Host Pathogen Interactions in Tuberculosis Laboratory, The Francis Crick Institute, NW1 1AT, London, UK.

[3] Lydia Becker Institute for Immunology and Inflammation, University of Manchester, M13 9PT, UK.

[4] Institut d'Investigació Germans Trias i Pujol, CIBER Enfermedades Respiratorias (CIBERES). Universitat Autònoma de Barcelona. 08916, Barcelona, Spain.

[5] Department of Pure and Applied Chemistry, University of Strathclyde, 295 Cathedral Street, G1 1XL, Glasgow, UK

*corresponding author

Lydia.tabernero@manchester.ac.uk

Table S-1. Equivalence of μg/ml and μM of non-commercial compounds used in this study (C13 and S-MGBs)

	**μg/ml**	**μM**	**FW (g/mol)**
**C13**	29.0	80	363
**S-MGB-362**	2.9	4	708.71
**S-MGB-363**	3.3	4	809.73

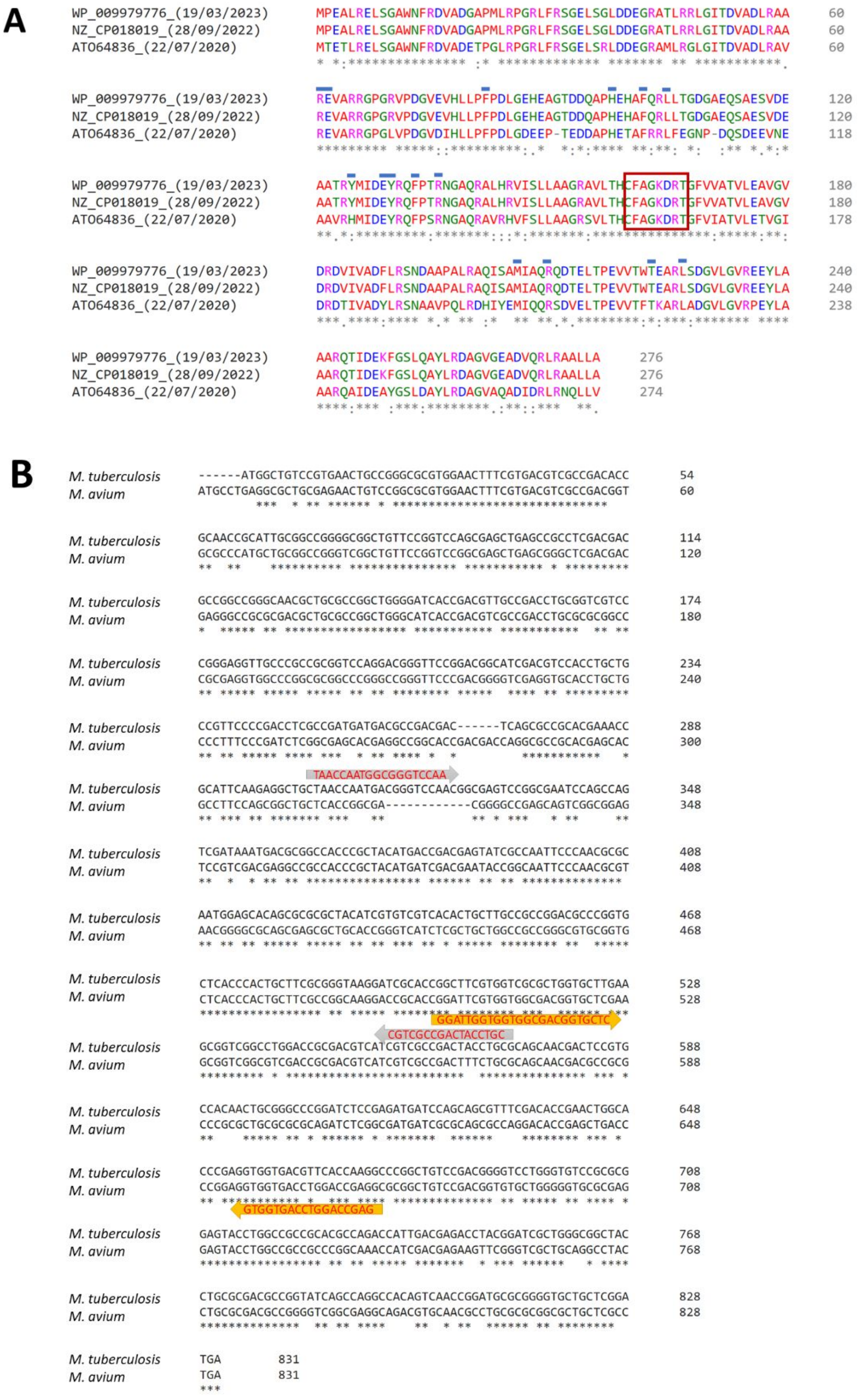
A
WP_009979776_(19/03/2023)
NZ_CP018019_(28/09/2022)
AT064836_(22/07/2020)
B
M. tuberculosis
M. avium
TAACCAATGGCGGGTCCAA
GGATTGGTGGTGGCGACGGTGCTC
CGTCGCCGACTACCTGC
GTGGTGACCTGGACCGAG

Figure S-1. Mav-ptpB is conserved. (A) Alignment of Mav-ptpB sequences (WP_009979776, NZ_CP018019, AT064836) shows at least 76% identity and 92% similarity. The boxed area indicates the active site signature. Under the blue line are shown conserved residues in the active site important for ligand binding [11,37]. In each column, "*" indicates identical residues, ":" conserved residues and "." semi-conserved residues. (B) Alignment of *mptpB* and *mav-ptpB* (WP_009979776) showing the primers binding site (grey for *M. bovis* BCG, yellow for *M. avium*). Reverse primers shows the reverse complement of the sequence. Alignments were prepared with ClustalX format citation with Omega[38].

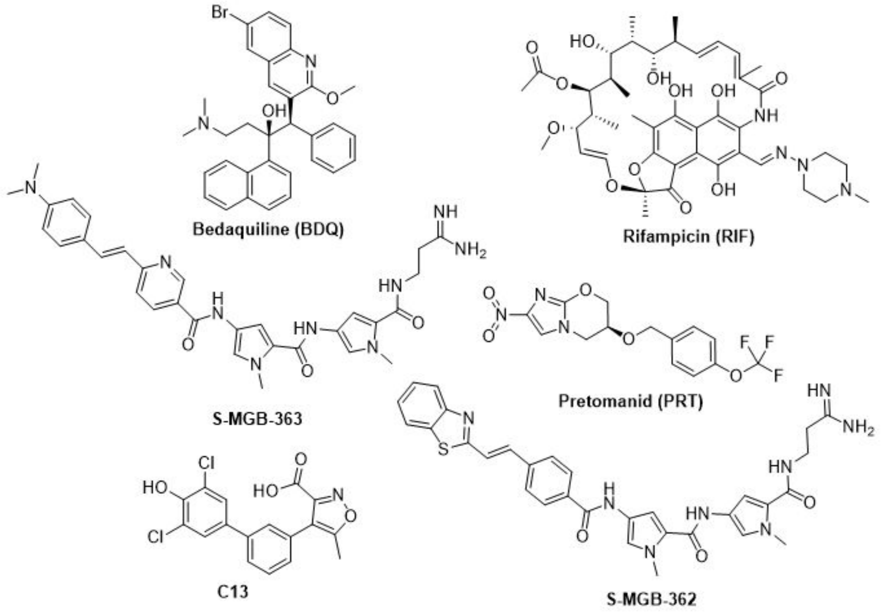

Figure S-2. Structure of the inhibitor C13, antibiotics RIF, BDQ and PRT, and S-MGBs used in this study.

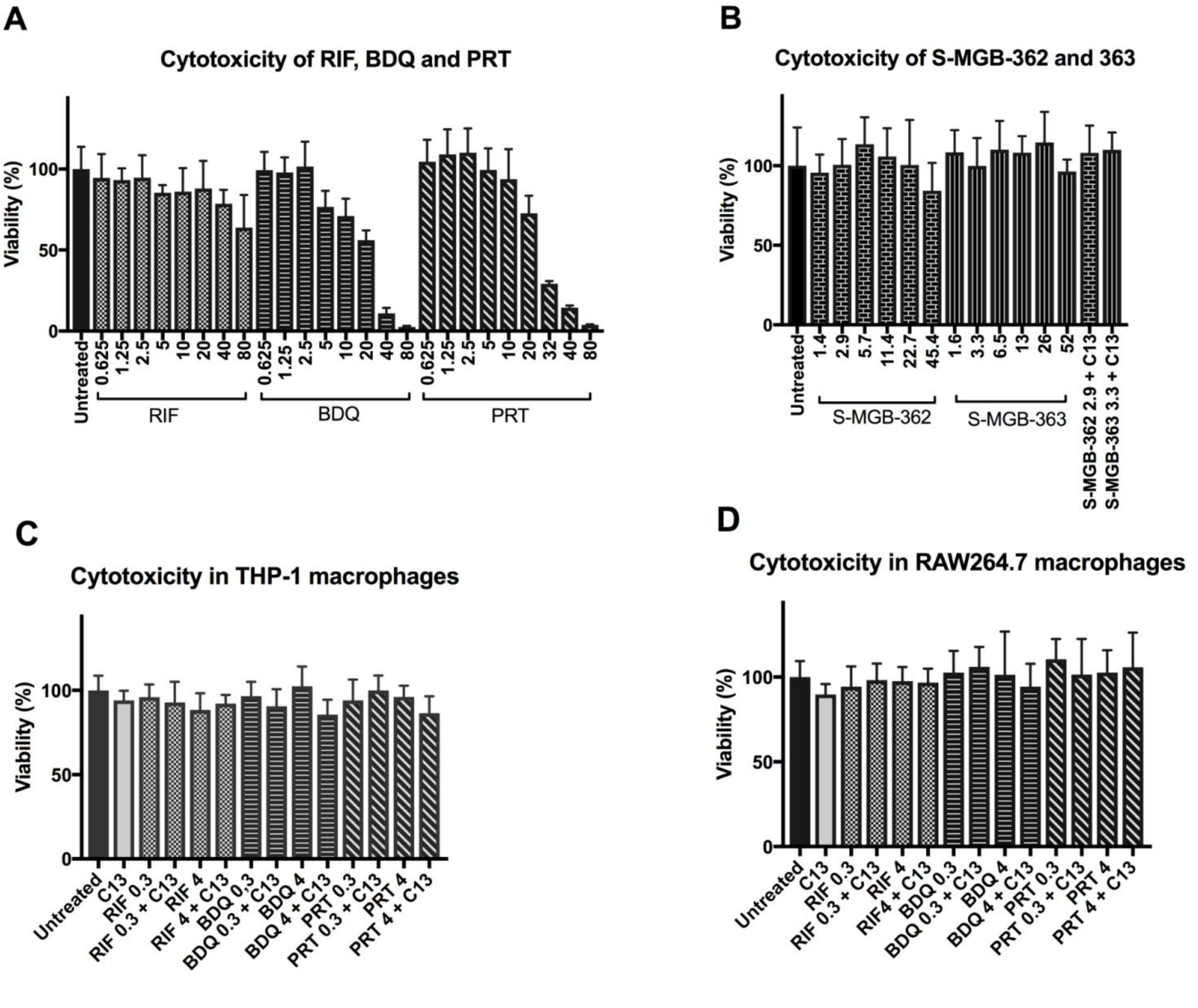

Figure S-3. Viability of THP-1 or RAW264.7 macrophages three days after exposing to antibiotics alone or in combination with C13 measured by MTT assay. (A) Dose-dependent assay to identify the maximum non-toxic dose of each antibiotic for RAW264.7 macrophages. (B) Dose-dependent assay for compounds S-MGB-362 and S-MGB-363 with THP-1 macrophages. Viability of THP-1 macrophages (C) and RAW264.7 macrophages (D) at the concentrations of antibiotics and combinations selected for this study. Data show the mean with SD of three technical replicates of at least two independent experiments. Concentrations are expressed in μg/ml except if stated otherwise.

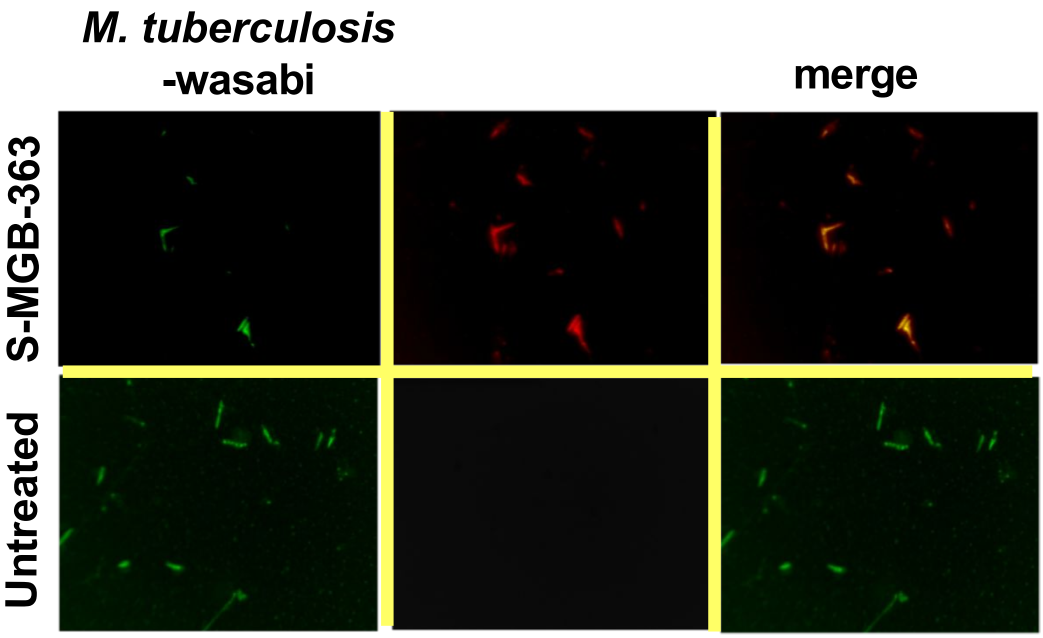

Figure S-4. Image showing that S-MGB-363 colocalizes to the bacterial DNA. Representative image showing *M. tuberculosis* expressing wasabi treated with S-MGB-363 (bacteria expressing wasabi is green, S-MGB-363 is shown in red)

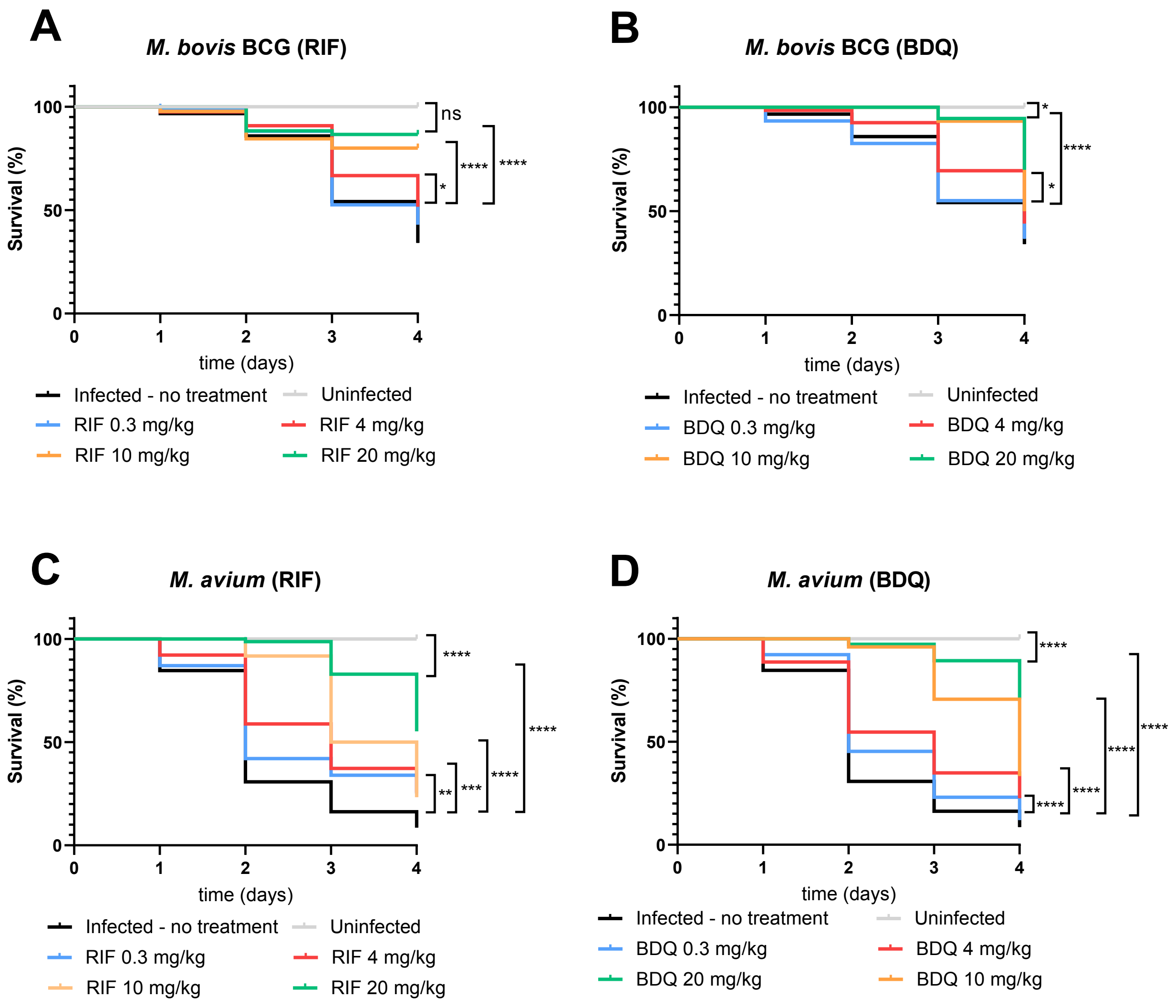

Figure S-5. Kaplan-Meier survival curves of *Galleria mellonella* larvae infected with BCG or *M. avium* and treated with C13 and or antimycobacterial drugs. Larvae (n=15 per group) were infected and treated in a 10 µl single injection. Survival was monitored every 24h over a period of 96h. Data are from a minimum of five independent experiments. Data shows the survival of *Galleria* infected with *M. bovis* BCG (A-B) or *M. avium* (C-D) when treated with different concentrations of RIF and BDQ**.** *$p < 0.05$, **$p < 0.01$, ***$p < 0.001$, ****$p < 0.0001$.

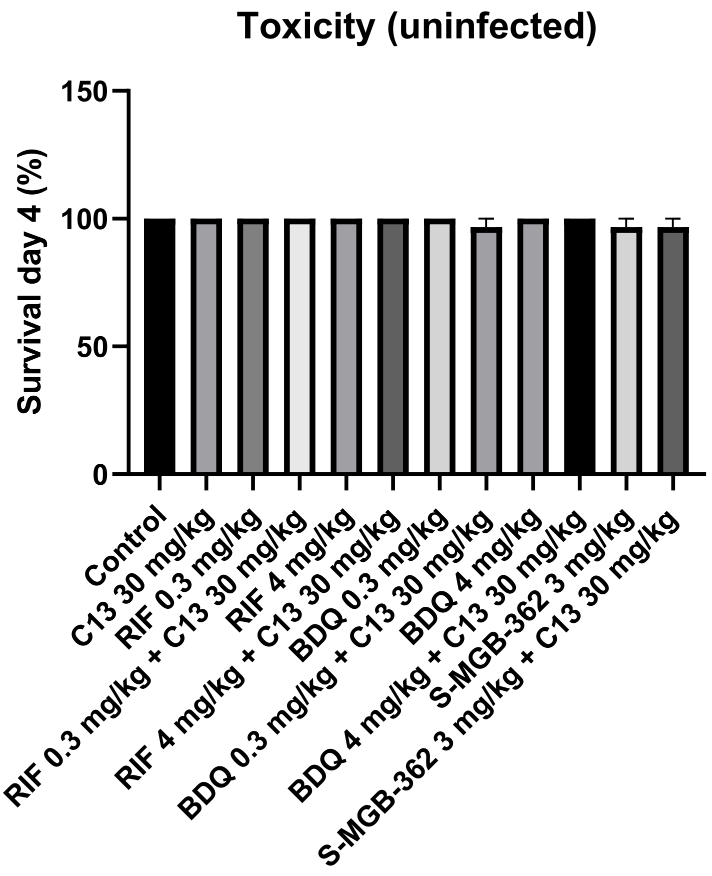

Figure S-6. Tolerability of compounds in *Galleria mellonella* uninfected larvae. Kaplan-Meier survival curves representing 15 larvae treated with a 10 µl single dose of C13 (30 mg/kg), RIF (0.3 or 4 mg/kg), BDQ (0.3 or 4 mg/kg), MGB (3 mg/kg) or combinations. Survival was monitored every 24h over a period of 96h. data are pooled from a minimum of two independent experiments.
